# A Transferable Lidar-Based Method to Conduct Contactless Assessments of Gait Parameters in Diverse Home-like Environments

**DOI:** 10.3390/s24041172

**Published:** 2024-02-10

**Authors:** Michael Single, Lena C. Bruhin, Aaron Colombo, Kevin Möri, Stephan M. Gerber, Jacob Lahr, Paul Krack, Stefan Klöppel, René M. Müri, Urs P. Mosimann, Tobias Nef

**Affiliations:** 1 Gerontechnology and Rehabilitation Group, ARTORG Center for Biomedical Engineering Research, University of Bern, 3012 Bern, Switzerland; michael.single@unibe.ch (M.S.); lena.bruhin@unibe.ch (L.C.B.); aaron.colombo@students.unibe.ch (A.C.); kevin.moeri@unibe.ch (K.M.); stephan.m.gerber@unibe.ch (S.M.G.); rene.mueri@unibe.ch (R.M.M.); urspeter.mosimann@insel.ch (U.P.M.); 2 University Hospital of Old Age Psychiatry and Psychotherapy, University of Bern, 3012 Bern, Switzerland; jacob.lahr@upd.ch (J.L.); stefan.kloeppel@upd.ch (S.K.); 3 Department of Neurology, Inselspital, University Hospital Bern, University of Bern, 3012 Bern, Switzerland

**Keywords:** gait analysis, Lidar, ambient sensors, person tracking, health monitoring

## Abstract

Gait abnormalities in older adults are linked to increased risks of falls, institutionalization, and mortality, necessitating accurate and frequent gait assessments beyond traditional clinical settings. Current methods, such as pressure-sensitive walkways, often lack the continuous natural environment monitoring needed to understand an individual’s gait fully during their daily activities. To address this gap, we present a Lidar-based method capable of unobtrusively and continuously tracking human leg movements in diverse home-like environments, aiming to match the accuracy of a clinical reference measurement system. We developed a calibration-free step extraction algorithm based on mathematical morphology to realize Lidar-based gait analysis. Clinical gait parameters of 45 healthy individuals were measured using Lidar and reference systems (a pressure-sensitive walkway and a video recording system). Each participant participated in three predefined ambulation experiments by walking over the walkway. We observed linear relationships with strong positive correlations ($R^{2}>0.9$) between the values of the gait parameters (step and stride length, step and stride time, cadence, and velocity) measured with the Lidar sensors and the pressure-sensitive walkway reference system. Moreover, the lower and upper 95% confidence intervals of all gait parameters were tight. The proposed algorithm can accurately derive gait parameters from Lidar data captured in home-like environments, with a performance not significantly less accurate than clinical reference systems.

## 1. Introduction

Gait abnormalities are associated with an increased risk of institutionalization, and affect almost one-third of community-dwelling older adults [1]. Furthermore, difficulties in gait and balance are associated with an increased risk of falls, which are linked to significant mortality in older adults [2]. In clinical practice, trained medical professionals commonly perform gait analysis through observational assessments. Video recordings and sensor technology may also be employed to enhance the precision and comprehensiveness of the evaluation. Traditional clinical assessments are conducted sparsely (e.g., once every few years or after an adverse event such as a fall) and, therefore, represent only a snapshot of the measured person’s health condition. More frequent measurements, or even continuous ones, are critical to obtaining a holistic picture of the person’s health status, since there can be significant inter-daily variability [3]. Additionally, objectivity is crucial in such gait analyses, since observer-based assessments may suffer from biases present in the human rater. One of the current reference sensor systems used to analyze gait is the pressure-sensitive walkway [4], which is limited to straight walks [5] and requires supervision by medical professionals [6,7]. Thus, there is a need for gait assessments in more natural environments both in and outside of the clinic, such as the homes of older adults or nursing homes, with an easy-to-use portable setup to record and analyze natural walking [8].

Another factor underlining the importance of gait assessments at home is the bimodality of human gait velocity, with one mode used for fast walking and another for slow and short walks [9]. Gait velocities derived from clinical evaluation settings do not align well with either mode observed in natural gait. Furthermore, it has been shown that assessments made in a supervised environment (i.e., at home) differ from clinical assessments due to the so-called white coat effect [10]. Continuous monitoring of clinical meaningful events in everyday environments also facilitates the detection of infrequent yet critical events such as falls and stumbles in older adults [11] or freezing of gait in people with Parkinson’s disease [12,13]. Comprehensive knowledge about these events, such as their frequencies and mechanisms, can support medical professionals in the diagnostic and therapeutic process, and can also improve our understanding of the associated pathologies [14,15,16]. Consequently, clinical studies increasingly include long-term measurements to capture natural walking behavior and potential critical events [17,18,19,20,21].

This need can be addressed with either wearable or ambient sensor technologies that allow a continuous spatiotemporal analysis of locomotion [22,23]. Ambient sensors used for gait analysis include the previously mentioned pressure-sensitive walkways, as well as motion capture systems [24,25]. The latter have limited applicability potential in older adults’ homes due to privacy concerns, physical challenges, and possible occlusion [25]. Wearable sensors, such as inertial measurement units (IMUs), measure the rotation and acceleration of the body part to which they are attached (e.g., the foot or ankle to measure gait) but require algorithmic processing to extract gait parameters [26], and compliance and user acceptance can be challenging [27,28].

There is, therefore, a strong need for an unobtrusive system that can measure gait precisely in a natural environment. Unobtrusive health monitoring can be defined as “using ambient sensor technologies to collect human health-related data without introducing any inconveniences to everyday life” [29]. A promising technology is light detection and ranging (Lidar), which has been used for object tracking in self-driving cars [30] and, more recently, for the accurate tracking of people in a natural environment [31,32]. Lidar-based sensors can cover a whole room without restricting movement, and have been used to measure gait, both in clinical environments [33,34] and in a home-like environment in a research apartment [35].

Thus, a natural next step is the application of Lidar technology in the form of a mobile setup that can be transported to where older adults live and naturally move around, and that can capture diverse settings and room layouts.

In this work, we describe a robust Lidar-based method to track human legs continuously in different home-like environments. This method can accurately quantify the gait parameters of people in different rooms while requiring only a minimal initial calibration effort, making it suitable for long-term measurements in everyday living settings in home-like environments. We hypothesized that the accuracy of gait parameters of healthy participants measured with Lidar in a home-like environment is not significantly different from that of gait parameters measured with the reference system, a pressure-sensitive walkway.

## 2. Materials and Methods

### 2.1. Participants and Setting

A convenience sample of 45 healthy individuals ranging in age from 22 to 72 years (mean 34.51 years, standard deviation (SD) 11.43 years) was recruited for this observational, cross-sectional study. The sample was gender-balanced, encompassing 25 women and 20 men. Participants were eligible for inclusion if they were at least 18 years of age and exhibited no walking impairments that could affect their regular daily activities. The study protocol was explained to each participant verbally, and written informed consent was obtained prior to participation. Two experiments were conducted: the first over three weeks (from November to December 2021) in a home-like instrumented apartment, the NeuroTec Loft, located at the Swiss Institute for Translational and Entrepreneurial Medicine (Inselspital Bern, Switzerland), measuring 30 individuals, and the second in a laboratory hallway in an office building (Bern, Switzerland), measuring 15 individuals.

### 2.2. Experimental Procedure

In the first experiment, each participant undertook three free ambulation exercises by walking over a pressure-sensitive walkway. Before initiating a measurement, participants were provided with detailed instructions. To ensure the participants understood the instructions, they were asked to perform a test run before each ambulation exercise. The free-walking experiments were recorded twice and conducted at a self-regulated pace, allowing participants to choose any pace that felt comfortable and natural to them. During the measurement, participants were asked to traverse the pressure-sensitive walkway without wearing shoes, eliminating the dampening effect of footwear as a confounding variable. To reduce further external influences that might have significantly affected the quality of the measured data, no person other than the subject being measured was in the vicinity of the sensor devices or walking around. For consistency, the starting position of every recorded walk was specified at the leftmost end of the pressure-sensitive walkway. Participants were asked not to leave the active area of the pressure-sensitive walkway at any point during the measurement to ensure the quality of the recorded data.

In the second experiment, each participant undertook one free ambulation experiment at a self-regulated pace in the hallway of the laboratory, with a test run before and two recordings of the ambulation. During the experiment, the participants were requested to traverse the hallway without wearing their shoes, and starting and ending positions were specified.

### 2.3. Data Collection Systems

**Figure 1:**
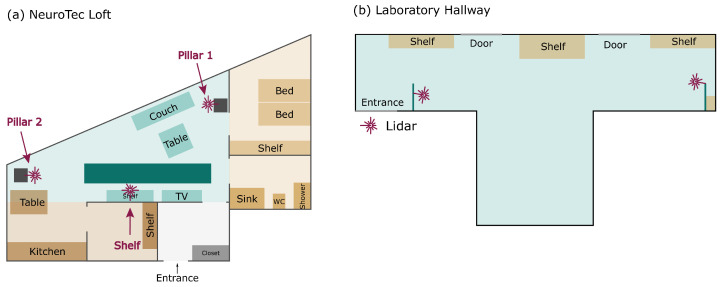
Schematic view of the room layouts of (**a**) the NeuroTec Loft and (**b**) the laboratory hallway, in which the experiments were carried out. The turquoise color stands for rooms where measurements took place, the purple star marks the locations of the Lidar sensors, and the dark turquoise color marks the pressure-sensitive walkway. The Lidar sensors were installed at a height that enabled tracking of a person’s shins.

In the first experiment, three Lidar sensors (UST-20LX-H01, Hokuyo Automatic Co., Ltd., Osaka, Japan) and a reference system, namely a pressure-sensitive walkway (GAITRite, CIR Systems Inc., Clifton, NJ, USA), were installed in the living room of the NeuroTec Loft. In the second experiment, two Lidar sensors were installed in a laboratory hallway. The layouts of the two experimental setups are shown in Figure 1.

Lidar sensors are based on time-of-flight technology, using a rotating infrared laser to measure distances. In this study, the sensors performed scans at a rate of 40 Hz, and each rotation spanned a 270-degree field, achieving an angular resolution of 0.125 degrees. The sensors were strategically placed at a height of 25 cm to track movements at the shin level effectively. This height was determined based on half the average sitting knee height of 53.5 cm, which is significantly higher than the average foot clearance of 2 cm ± 1 cm [36,37]. This height aligns with similar studies, in which the Lidar sensors were typically positioned between 20 cm and 40 cm above the ground [33,35,38,39]. A basic Python client–server application was developed to read the Lidar samples from the sensors and transfer them via the network to a database using the sensor recording software system [40].

To measure spatial and temporal gait parameters, a pressure-sensitive walkway was used as a reference system in the first experiment. The specific pressure-sensitive walkway model employed had an active measurement length of 4.88 m and a width of 0.61 m. The data gathered from the system were sampled at a frequency of 80 Hz and analyzed using the GAITRite software (version 4.89H9). The following gait parameters were obtained from the analysis: average step time, average cycle time, total ambulation time, cadence per minute, average velocity, and average step length.

In addition to the pressure-sensitive reference system, a validated set of IMU sensors (Physilog5, Gait Up, Lausanne, Switzerland) served as the reference system in the second experiment. Each such sensor was mounted on a participant’s foot, recording its acceleration and orientation at 128 Hz. The analysis of gait was conducted using the Gait Up LAB software (Gait Up, Switzerland), version 1.0.1. The gait metrics derived from these wearable sensors for assessment included step time, stride time, and cycle time.

### 2.4. Gait Parameter Computation

**Figure 2:**
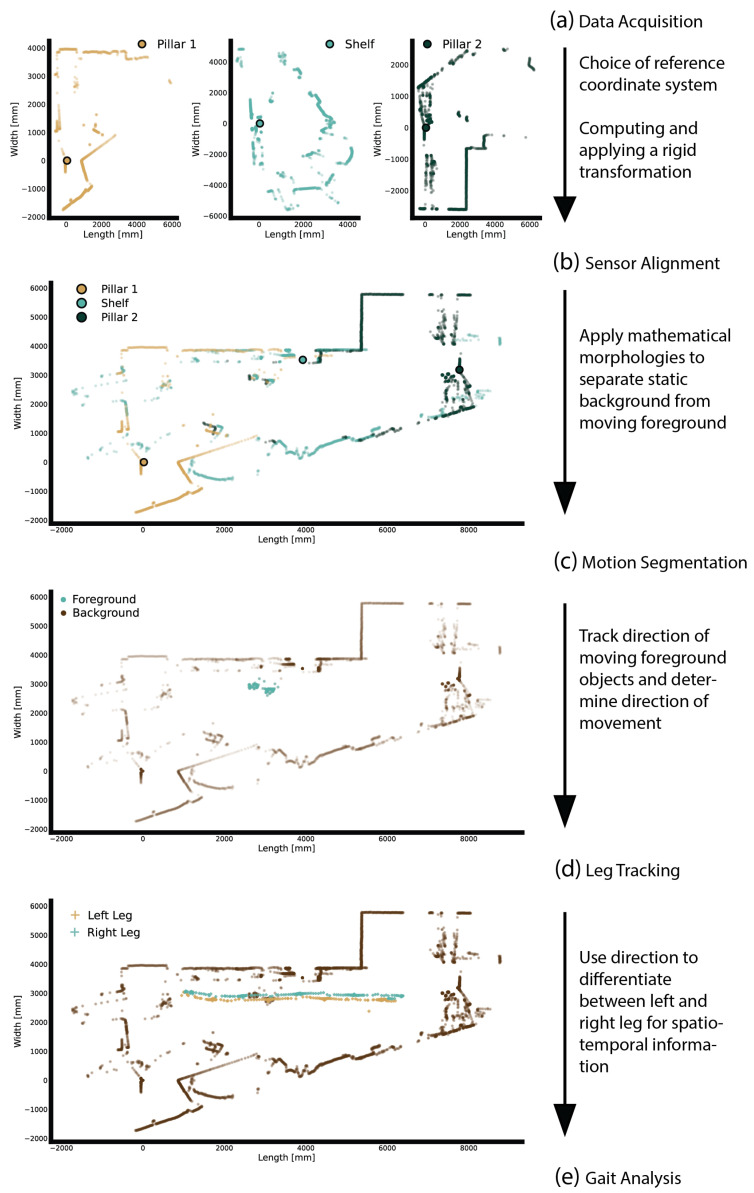
Illustration of the stages of the Lidar-based leg tracking algorithm developed to analyze human gait. (**a**) Raw Lidar signals are transformed from polar coordinates to Cartesian coordinates. (**b**) The resulting Lidar frames are aligned by choosing a reference coordinate system (Pillar 1), and then computing and applying a rigid transformation to the remaining sensors. (**c**) Motion segmentation is performed to separate the static background (e.g., props, walls) from the moving object (e.g., the legs of a walking person). (**d**) Spatiotemporal clustering is performed for the foreground to distinguish between the left and right legs. (**e**) Gait analysis is performed by calculating velocity profiles from the tracking data.

The core idea of the proposed algorithm is that a temporally coherent distance measurement can be used to perform motion segmentation and thus track human movements. Initially, the raw Lidar signals were transformed from polar coordinates (i.e., angles and distances) to Cartesian coordinates (i.e., positions in a metric coordinate system). The stages of the Lidar-based gait parameter computation algorithm included sensor alignment, motion segmentation, leg tracking, and gait analysis (Figure 2).

#### 2.4.1. Sensor Alignment

In the process of aligning the Lidar data, we aimed to make them temporally and spatially comparable. This involved synchronizing the measurement timestamps to ensure a uniform sampling rate. We achieved this by resampling the data using linear interpolation. Based on the assumption that all Lidar sensors operate within the same metric space, and thus ensuring consistency in sizes and shapes across different sensors, we established the spatial alignment of the Lidar coordinate systems by employing a rigid transformation method. The first step in this process was to choose one Lidar sensor as the reference coordinate system.

Subsequently, we determined a rigid transformation for each of the other sensors in relation to this reference system. This required identifying two pairs of corresponding points and solving a linear system of equations. The best point-to-point correspondences were selected by applying a SIFT feature descriptor to the binary image representation of the Lidar scans [41]. The final rigid transformations (Equation (A7)) were computed based on the determined point correspondences and applied to all measured Lidar samples to achieve spatial alignment. A detailed step-by-step derivation of the rigid transformation from point correspondences is provided in Appendix B. The resulting aligned Lidar scans for each timestamp will henceforth be referred to as a frame.

#### 2.4.2. Motion Segmentation

**Figure 3:**
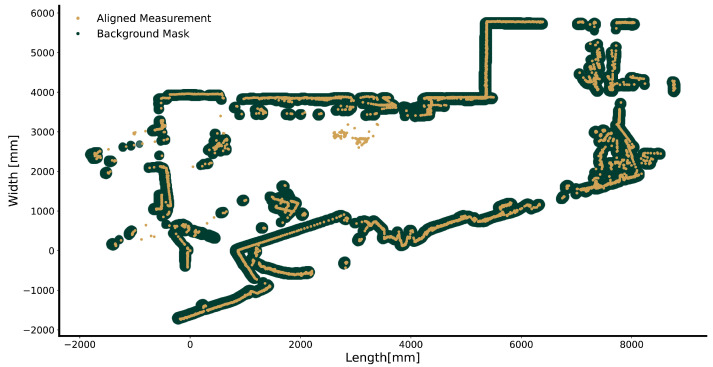
Illustration of the aligned Lidar measurements (brown) and the background mask (dark green).

To distinguish between static features (e.g., furniture, walls) and motion in the measurements, we computed a mask that indicates which measurement location belongs to the background. This was accomplished using a stochastic approach in which we computed the average of 200 randomly chosen aligned frames. We enlarged the pixels of the resulting average image and removed small holes by applying the morphological closing operator with a spherical structural element (Figure 3). Every measurement in an aligned frame underwent the following test: if a measurement intersected the background mask, the measurement was marked as background and otherwise as foreground. It is noteworthy that the motion segmentation technique does not require an initial static measurement. More details related to mathematical morphology can be found in Appendix A.

#### 2.4.3. Leg Tracking

In this phase, we temporally clustered the previously extracted foreground samples and identified the leg to which each cluster pertained. Initially, noise in the foreground data was reduced by relying on an agglomerative hierarchical clustering method [42]. This approach enabled us to use tailored distance metrics and specify the expected number of clusters. Based on the assumption that only the person to be measured was moving during a recording, the number of expected clusters was hard-coded as four (one cluster for each leg and two to account for any similar noise). To calculate the distances between data points, we used the Euclidean distance formula. The linkage distance threshold, at which clusters were no longer merged, was set to 0.3 m to resemble the distance between two legs. Finally, we discarded any clusters that had fewer than 5 or more than 50 samples. Based on the number of remaining clusters, the following three scenarios needed to be considered:

1. If exactly two clusters remained, the *k*-means algorithm was employed to determine the new centroids of these clusters.
2. If fewer than two clusters remained, this indicated a failure to track one or both legs. This was addressed by predicting the missing leg’s position by analyzing its location in preceding frames. This involved calculating the finite difference between a frame and the one before it, and using these trajectory patterns to estimate the leg’s current position based on its last known position. These estimated positions were used as the centroids in the current frame.
3. If more than two clusters remained, clusters in close proximity were merged until a maximum of two remained. The merging criterion was based on the distance between their centroids, calculated as the aggregate of all points within each cluster. If the distance between two centroids was less than or equal to 25 cm (i.e., approximately the maximum diameter of a leg), they were considered too close and were merged. After merging, a new centroid for the resulting cluster was calculated.

The clusters were identified with the left and right legs by analyzing the calculated centroids. The initial classification was performed by assessing the angle between the direction vector and the leg position vector, which was anchored to the measured person’s body center. In successive frames, we assigned the same label to each shin position based on its proximity to the closest identified shin position from the previous frame.

#### 2.4.4. Gait Analysis

**Figure 4:**
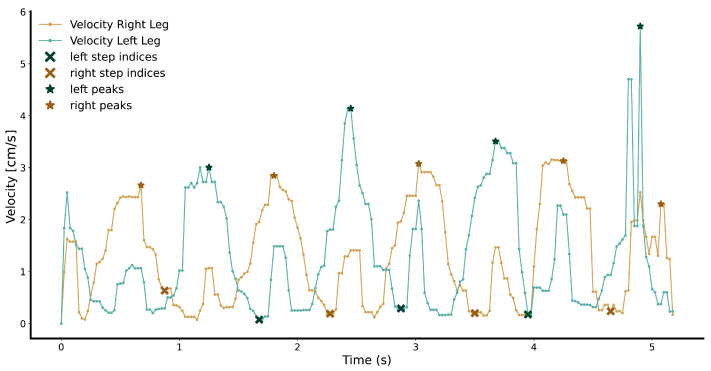
Illustration of the velocity profiles of the tracked legs. The velocity profile of the right leg is plotted in brown colors, and the velocity profile of the left leg is plotted in blue–green colors. In both profiles, peaks are marked using star symbols, and steps of the corresponding leg using crosses.

To compute gait parameters, the extracted leg positions were used to calculate velocity profiles for both legs (Figure 4).

Two median filters were used to remove outliers from the velocity curves. Then, hard-coded peak detectors were used to find major velocity peaks corresponding to the swing period of a step, minor velocity peaks corresponding to the bending of the ankle during the stance phase, and velocity valleys corresponding to near-zero velocities during the stance phase. Subsequently, the median point of each valley after a major and before a minor peak was calculated to represent the heel strike. These heel strike events were used to calculate temporal (step and stride time), spatial (step and stride length), and spatiotemporal (cadence and velocity) gait parameters (Table 1). The spatial coordinates of steps were extracted by finding the leg positions at the timestamp of the heel strike events.

The source code of the Lidar-based gait computation algorithm is freely available on GitHub, along with detailed documentation and a gait dataset [43].

### 2.5. Statistical Analysis

A comprehensive statistical comparison was conducted between the gait parameters computed using the Lidar-based method and the measurements acquired from the reference system (i.e., the pressure-sensitive walkway). More specifically, the two measurement systems were analyzed by comparing gait parameters calculated from different free ambulation experiments for the same participant. To establish an adequate comparison, we confined the statistical analysis to those Lidar measurements that occurred within the time frame of the pressure-sensitive walkway recordings.

Initially, descriptive statistics of all gait parameters were calculated for the Lidar sensors and the pressure-sensitive walkway. Scatter plots of the distributions in step lengths and velocities were qualitatively compared between the two measurement methods.

Subsequently, the significance of the difference between the Lidar and pressure-sensitive walkway measurements was evaluated by performing paired two-sample *t*-tests for all matching gait parameters. We hypothesized that the mean values of matching gait parameters between the Lidar and pressure-sensitive walkway measurements would not be significantly different (significance level α = 0.01).

Next, a simple linear regression was applied to the mean values of the gait parameters from all ambulation experiments to analyze the relationship between the two measurement systems. The necessity of a linear regression model was validated by performing an *F*-test. This test incorporated the residual sum of squares for each paired set of measured and computed gait parameters. The coefficient of determination, $R^{2}$, was calculated for each matching gait parameter to help clarify the accuracy of the linear models. Additional error metrics, such as the standard error, the mean square error, and the mean difference, were calculated for all gait parameters. The correlation between the Lidar measurements and the pressure-sensitive walkway measurements of matching gait parameters was quantified by computing the Pearson correlation coefficient, denoted by *r*. To interpret the correlation results qualitatively, we applied the terminology proposed by Schober et al. [44].

Finally, the measured matching gait parameters were qualitatively examined using Bland–Altman plots. These plots were used to identify the presence of proportional and constant biases in the data within the confidence intervals, as well as to determine the agreements between the matching gait parameters measured using the pressure-sensitive walkway and the Lidar sensors.

The data assessed in the hallway experiment were reported in the form of descriptive statistics of the Lidar-based method and the IMUs. This included box plot visualizations of the measured gait parameters (step time, stride time, and cadence).

## 3. Results

### 3.1. Descriptive Analysis of Step Length and Velocity

In this study, a total of 90 ambulation experiments were conducted with 30 participants (each performed three walks). The proposed Lidar-based method detected the correct number of performed steps (594).

**Figure 5:**
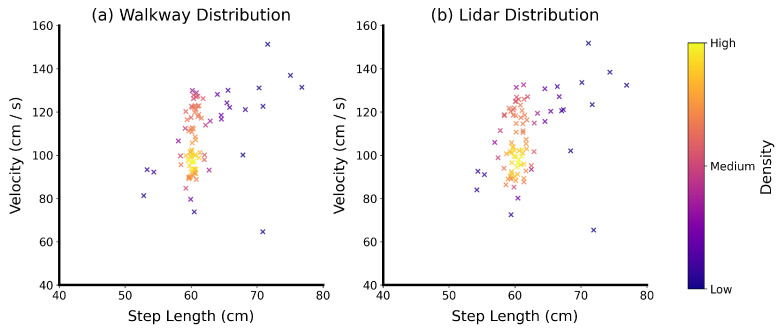
Distributions of step lengths and velocities across all ambulation experiments for (**a**) the pressure-sensitive walkway measurements and (**b**) the Lidar measurements. To provide a qualitative comparison between the two, their density and shape are visualized. Colors are used to highlight the high-density regions.

The distributions of step lengths and velocities show similar characteristics for both the Lidar and walkway methods (Figure 5). Both attain their highest density in the same region, which can be seen in the yellow cluster at a step length of 60 cm and a velocity of 100 cm/s. Furthermore, the distributions have similar shapes, with the Lidar distribution showing a slightly larger spread.

The mean velocity of ambulation measured by the pressure-sensitive walkways was 107.3 cm/s (SD = 15.8 cm/s), ranging between 64.6 cm/s and 151.3 cm/s, and the mean velocity measured using Lidar was 107.5 cm/s (SD = 16.1 cm/s), ranging between 65.4 cm/s and 151.8 cm/s. The mean step length measured with the pressure-sensitive walkway was 61.4 cm (SD = 3.8 cm), ranging between 52.8 cm and 76.8 cm, and the mean step length measured with Lidar was 61.5 cm (SD = 3.9 cm), ranging between 54.2 cm and 76.9 cm.

### 3.2. Comparison of Gait Parameters between Lidar Sensors and Pressure-Sensitive Walkway

The results of the paired two-sample *t*-tests showed that there was no significant difference between the gait measurements obtained using the pressure-sensitive walkway and the Lidar sensors (Table 2), except for the stride length. All mean values and standard deviations were similar between the two methods. The effect size of the analyzed gait parameters was small (d<0.2), except for stride length.

All gait parameters showed a strong positive correlation between the measurements assessed with the Lidar sensors and the pressure-sensitive walkway, all with small standard errors, root-mean-square errors (RMSEs), and mean difference values, except for stride length (Table 3). The RMSE for stride length was approximately 2.9 cm. All gait parameters exhibited Pearson correlation coefficients greater than 0.94. Moreover, for all gait parameters except stride length, the lower and upper 95% confidence intervals were tight.

**Figure 6:**
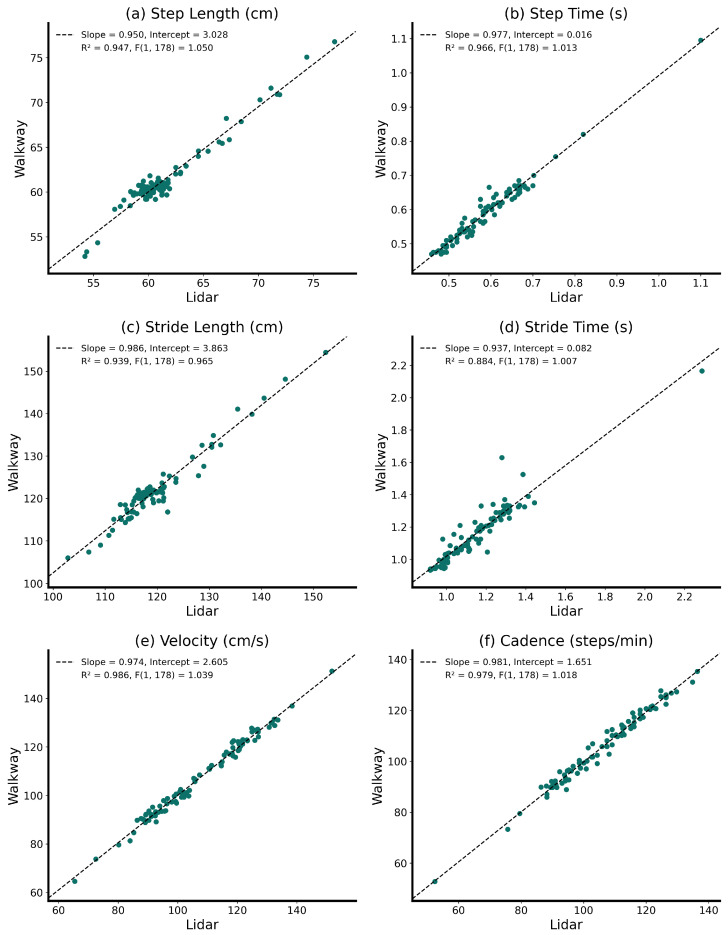
Linear relationships between gait parameters assessed using the pressure-sensitive walkway and using the Lidar sensors. For each plot, slope and intercept values of the fitted linear model, the determination coefficients, $R^{2}$, and the F-scores are provided.

On the basis of the *F*-statistics, we found significant linear relationships between gait parameters measured using the Lidar sensors and the pressure-sensitive walkway (Figure 6), with slopes close to one. All parameters showed a high coefficient of determination (i.e., $R^{2}>0.9$).

**Figure 7:**
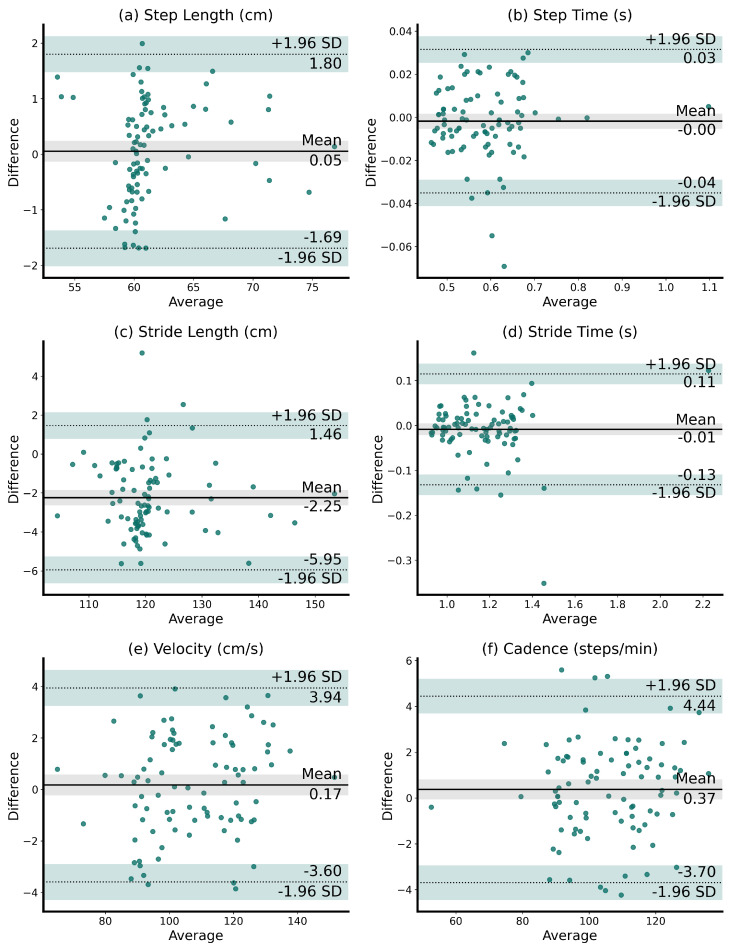
Bland–Altman plots demonstrating the agreement between the matching gait parameters from the Lidar and the pressure-sensitive walkway measurements. The average measurements from the two methods are plotted on the *x*-axis, and the differences between them on the *y*-axis. No proportional bias and a close-to-zero constant bias were found. The limits of the agreements, encompassing 95% of all differences between measurements, exhibited acceptable values.

According to the Bland–Altman plots shown in Figure 7, we observed an agreement between the gait parameters measured using the pressure-sensitive walkway and the Lidar sensors.

The step length was between −1.69 cm and 1.80 cm, the step time was between −0.04 s and 0.03 s, the stride length was between −5.95 cm and 1.46 cm, the stride time was between −0.13 s and 0.11 s, the velocity was between −3.60 cm/s and 3.94 cm/s, and the cadence was between −3.70 steps/min and 4.44 steps/min. Additionally, the identified biases were minimal, highlighting no proportional bias and approximately zero constant bias (i.e., the difference between 0 and the black mean line was small, and no trend in the data distribution was seen). The limits of the agreements, encompassing 95% of all differences between measurements, exhibited acceptable values and only a small number of outliers. The variability of the difference was consistent and low across different means.

### 3.3. Comparison between Lidar and Accelerometer Measurements

**Figure 8:**
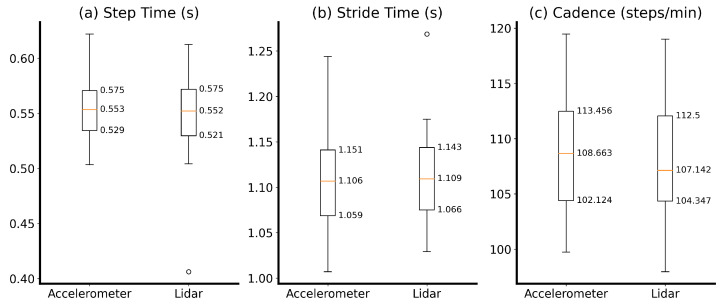
Descriptive statistics of (**a**) the step time, (**b**) the stride time, and (**c**) the cadence measured with accelerometers and the Lidar-based method. Orange lines represent the median, the box indicates interquartile ranges, and circles show outliers.

In total, 15 individuals were measured walking along the laboratory hallway while wearing two accelerometers. Descriptive statistics of the measurements are shown in Figure 8.

The mean step time recorded with the accelerometers was 0.556 s (SD = 0.032 s), ranging between 0.504 s and 0.622 s, and the mean step time measured with the Lidar was 0.545 s (SD = 0.047 s), ranging between 0.406 s and 0.613 s. The mean stride time from the accelerometers was 1.112 s (SD = 0.064 s), ranging between 1.007 s and 1.244 s, and the mean stride time from the Lidar sensors was 1.113 s (SD = 0.059 s), ranging between 1.029 s and 1.269 s. The mean cadence measured with the accelerometers was 108.723 steps per minute (SD = 5.752), ranging between 99.733 and 119.477, and the mean cadence value from the Lidar sensors was 107.915 steps per minute (SD = 6.009), ranging between 97.959 and 119.008.

## 4. Discussion

In this work, we developed a robust Lidar-based method to track human legs continuously in different home-like environments. The developed method requires neither calibration to align its Lidar scans nor static background measurement to distinguish between foreground and background. In line with the hypothesis, the study provided evidence showing that the mean values of matching gait parameters derived from the Lidar-based and pressure-sensitive walkway measurements exhibited no significant differences.

Overall, we observed linear relationships with strong positive correlations between the gait parameters (step length, step time, stride length, stride time, velocity, and cadence) measured with the Lidar sensors and the pressure-sensitive walkway reference system. The accuracy of the calculated temporal and spatial gait parameters was consistent with previous studies [26,35,45]. Moreover, the Lidar-based velocity–step length distributions closely resembled those measured using the pressure-sensitive walkway. As such distributions have been shown to indicate health changes in older adults, this finding suggests the developed Lidar-based method can serve as an objective digital measurement tool to detect such health changes [46]. Finally, the agreements between the Lidar sensors and the pressure-sensitive walkway resulting from the Bland–Altman plots were high, except for stride length. Nevertheless, the Pearson correlations were very high for all gait parameters, suggesting that different calculation methods might have led to the discrepancy in stride length. Also, no proportional bias and a close-to-zero constant bias were found, implying the absence of a systematic error.

Our method was developed in accordance with the principles outlined in the systematic review by Toro et al., with particular emphasis on ensuring validity and repeatability in gait assessment tools, as well as mitigating biases emerging from subjective elements in the assessment process [47]. In comparison with gait assessments conducted with wearable devices like accelerometers, where compliance has often been suboptimal in patients with neurodegenerative disease or older adults, our methods offer the advantage of easy installation without requiring direct interaction with the patient [27,28]. Moreover, determining step length using accelerometer data is known to be a challenging task [48]. This contrasts with the more straightforward approach of direct distance measurement techniques, such as those employed in the Lidar technology used in our method. Regarding the measurement errors, the determined ranges of velocity, step time, and length, and cadence values were confirmed by referring to findings from the literature [35,49,50]. Therefore, these results provide evidence that the Lidar-based method extracted gait parameters with high consistency and reliability. Additionally, Lidar-based gait assessments were shown to be a reasonable method to measure gait parameters in a clinically acceptable accuracy range. Notably, an accurate assessment of these results was achieved without intrusive installation or calibration of the sensing devices. Considering the consistency in the measurements, the proposed method is promising not only as an ambient technology but also as a reliable solution for ongoing home monitoring of older adults [51]. Furthermore, due to the portable nature of our Lidar system, it can be used to perform long-term gait measurements in the natural environments of older adults, ultimately leading to a more objective medical assessment. This can be further underlined with the findings obtained in the laboratory hallway experiment, where we demonstrated that Lidar sensor measurements lead to high correlation with temporal gait parameters measured with IMUs (i.e., step time, stride time, and cadence).

The importance of long-term gait monitorization methods becomes even clearer when considering the findings of Van Ancum et al. [9], which showed that gait speed measured in clinical settings does not fully reflect natural gait. Considering the crucial role of gait parameters in evaluating age-related diseases like Parkinson’s, implementing a Lidar-based gait monitoring system in residential homes or assisted living environments would enable monitoring over a substantial part of the day, and thus might better reflect the true gait speed.

One observed shortcoming of the proposed algorithm is that the calculated spatial gait parameter (i.e., stride length) performed worse than its temporal counterpart (stride time), although the linear models demonstrated high coefficients of determination, signifying that they could explain more than 90% of the variations. This difference in accuracy might be a direct consequence of the hard-coded peak detector used to identify steps from the velocity profiles. An alternative explanation for measuring slightly too short stride lengths could also be attributed to the geometric considerations of Lidar placement at shin height: as the height of measurement increases, the recorded stride lengths appear shorter. Although optimizing the algorithm detecting peaks was not the focus of the present work, the problem could be overcome by using an adaptive peak detector based on dilated convolutional neural networks [52].

This study only used gait measurements from healthy participants, which potentially restricts its generalizability, especially considering the significant differences in gait patterns found in individuals with neurological disorders or in older adults [22]. Hence, subsequent studies should include people with walking impairments or who belong to an older demographic with a raised falling risk.

As a next step, multi-person tracking could be included using techniques akin to Kalman filters [53]. Additionally, Lidar-based gait analysis could be applied to behavioral assessments for common neurological and psychiatric diseases (e.g., depression, Alzheimer’s, and Parkinson’s disease) as a means of monitoring cognition, daily fluctuations of medication, or night and day rhythm.

## 5. Conclusions

This study presents a novel Lidar-based method that can be used to measure gait parameters without a calibration step. We demonstrated that the accuracy of the proposed method is comparable with clinical reference systems, such as a pressure-sensitive walkway. The gait analysis algorithm filters features of interest and simultaneously attenuates noise to make the Lidar-based method easily transferable to new settings convenient for hospital patients and older adults. Finally, these unobtrusive ambient sensors represent a promising technology with high accuracy in measuring gait parameters outside the hospital environment, and could be used to conduct longitudinal studies with broad application in the common geriatric, neurodegenerative, and psychiatric indications for monitoring fluctuating states, disease progression, or treatment effects.

## Figures and Tables

**Table 1:** Temporal and spatial gait parameters and how they were computed.

Gait Parameter	Computation (per Ambulation)
Step Length (cm)	The distance between two consecutive heel strike events of contralateral legs found by extracting leg position at the time of the two heel strikes
Step Time (s)	The time between two consecutive steps of contralateral legs
Stride Length (cm)	The distance between two consecutive steps of the same leg
Stride Time (s)	The time between two consecutive steps of the same leg
Cadence (1/min)	The number of steps per minute
Velocity (cm/s)	The distance in centimeters per second

**Table 2:** Paired two-sample *t*-tests of the gait parameters. The adjusted *p*-value is in boldface if it fell at or below α=0.01. No significant differences were found between the gait measurements on the pressure-sensitive walkway and the Lidar sensors at the specified significance level, except for the stride length. The degree of freedom for all tests was 89. Furthermore, the mean and standard deviation values were comparable across both measurement devices. A small size effect was observed, as indicated by Cohen’s *d* statistic.

Gait Parameter	Walkway		Lidar		t(90)	*p*	*d*
	M	SD	M	SD			
Step Length (cm)	61.433	3.769	61.487	3.861	0.570	0.570	0.014
Step Time (s)	0.586	0.090	0.584	0.091	−1.020	0.311	0.020
Stride Length (cm)	121.791	7.607	119.544	7.473	−111.279	<0.01	0.296
Stride Time (s)	1.169	0.180	1.160	0.181	−11.320	0.190	0.048
Velocity (cm/s)	107.289	15.834	107.462	16.141	0.854	0.395	0.011
Cadence (steps/min)	105.210	14.269	105.581	14.397	1.695	0.094	0.026

**Table 3:** Pearson correlation statistics of simple linear regression models between Lidar and pressure-sensitive walkway measurements. The mean differences, the standard error, and the root-mean-square error were computed between the Lidar and pressure-sensitive walkway measurements for all gait parameters. The 95% confidence interval for all gait parameters was computed to obtain a qualitative understanding of the Lidar measurements. Number of walks = 90; CI = confidence interval; LL = lower limit; UL = upper limit; mean = mean difference between the Lidar and pressure-sensitive walkway values; RMSE = root-mean-square error; SE = standard error; and r is the Pearson correlation coefficient.

Gait Parameter	*r*(90)	Mean	SE	RMSE	95% CI		*p*
					LL	UL	
Step Length (cm)	0.973	0.054	0.024	0.888	−0.13	0.24	<0.001
Step Time (s)	0.983	−0.002	0.020	0.017	−0.01	0.0	<0.001
Stride Length (cm)	0.969	−2.246	0.027	2.928	−2.64	−1.85	<0.001
Stride Time (s)	0.940	−0.009	0.036	0.063	−0.02	0.0	<0.001
Velocity (cm/s)	0.993	0.173	0.012	1.921	−0.23	0.58	<0.001
Cadence (steps/min)	0.990	0.371	0.015	2.100	−0.06	0.81	<0.001

## Data Availability

The raw data that support the findings of this study are not openly available due to reasons of sensitivity. The associated processed raw data are available from the corresponding author, T.N., upon reasonable request.

## Abbreviations

The following abbreviations are used in this manuscript:

IMU	Inertial measurement unit
Lidar	Light detection and ranging
M	Mean value
RMSE	Root-mean-square error
SD	Standard deviation
SE	Standard error

## Appendix A. Mathematical Morphologies

Mathematical morphology is a filtering technique commonly employed in computer vision to extract the geometric features of objects contained in digital images [54]. The basic idea of morphological filtering involves processing a signal, *f*, using a structural element, *g*, a basic shape that resembles the features of interest in *f*. This filtering relies on two fundamental operators, dilation (⊕) and erosion (⊖). Mathematically, dilating *f* by the structural element *g* is defined as

(A1) $$\left(f⊕g\right)\left(t\right)=\max\left(f\left(z-x\right)+g\left(x\right)|x\in g,\left(z-x\right)\in f\right).$$

Eroding *f* by *g* is defined as

(A2) $$\left(f⊖g\right)\left(t\right)=\min\left(f\left(z+x\right)-g\left(x\right)|x\in g,\left(z+x\right)\in f\right).$$

The shape and size of the structural element typically depend on the target signal *f* and determine the performance of the morphology-based processing. The purpose of the dilation operation is to expand or smooth peaks in the signal, whereas erosion contracts or sharpens troughs. The compound operations, the opening (∘) and the closing (•), are defined as sequential combinations of the dilation and the erosion operations [54]. More precisely, applying an erosion followed by a dilation is denoted as the opening operation,

(A3) $$f\circ g=\left(f⊖g\right)⊕g,$$

and applying a dilation followed by an erosion is denoted as the closing operation,

(A4) $$f•g=\left(f⊕g\right)⊖g.$$

## Appendix B. Rigid Transformations

The following section describes how the rigid transformations that were used to align the Lidar frame were computed. For two corresponding pairs of 2D points, (p1,q1) and (p2,q2), we calculated the rigid transformation as a homogeneous transformation matrix. In terms of homogeneous coordinate systems, every rigid transformation can be described as

(A5) $$\begin{array}{cc} \underset{q}{\underset{︸}{\left(\begin{array}{c} q_{x}\\ q_{y}\\ 0 \end{array}\right)}}\; & =\underset{T}{\underset{︸}{\left(\begin{array}{ccc} a & -b & c\\ b & a & d\\ 0 & 0 & 1 \end{array}\right)}}\underset{p}{\underset{︸}{\left(\begin{array}{c} p_{x}\\ p_{y}\\ 1 \end{array}\right).}} \end{array}$$

The parameters *a* and *b* define the rotation, and *c* and *d* define the translation. The relation between these points, $p=(p_{x},p_{y})$ and $q=(q_{x},q_{y})$, and the rigid transform matrix, T, expresses a one-point correspondence, which can be rewritten in the following form:

(A6) $$\begin{array}{cc} \left(\begin{array}{c} q_{x_{i}}\\ q_{y_{i}} \end{array}\right)\; & =\left(\begin{array}{cccc} p_{x_{i}} & -p_{y_{i}} & 1 & 0\\ p_{y_{i}} & p_{x_{i}} & 0 & 1 \end{array}\right)\left(\begin{array}{c} a\\ b\\ c\\ d \end{array}\right). \end{array}$$

To extend this expression to a two-point correspondence, we stacked the $\left(q_{x_{i}},q_{y_{i}}\right)$ values and also added two rows to the matrix on the right-hand side as follows:

(A7) $$\begin{array}{cc} \underset{y}{\underset{︸}{\left(\begin{array}{c} q_{x_{1}}\\ q_{y_{1}}\\ q_{x_{2}}\\ q_{y_{2}} \end{array}\right)}}\; & =\underset{A}{\underset{︸}{\left(\begin{array}{cccc} p_{x_{1}} & -p_{y_{1}} & 1 & 0\\ p_{y_{1}} & p_{x_{1}} & 0 & 1\\ p_{x_{2}} & -p_{y_{2}} & 1 & 0\\ p_{y_{2}} & p_{x_{2}} & 0 & 1 \end{array}\right)}}\underset{x}{\underset{︸}{\left(\begin{array}{c} a\\ b\\ c\\ d \end{array}\right)}}. \end{array}$$

The rigid transformation, T, is computed by determining the parameters in x by solving

(A8) $$\begin{array}{c} x=A^{-1}y. \end{array}$$

## References

[1] Verghese J., LeValley A., Hall C.B., Katz M.J., Ambrose A.F., Lipton R.B. (2006). Epidemiology of gait disorders in community-residing older adults. J. Am. Geriatr. Soc..

[2] Osoba M.Y., Rao A.K., Agrawal S.K., Lalwani A.K. (2019). Balance and gait in the elderly: A contemporary review. Laryngoscope Investig. Otolaryngol..

[3] Schütz N., Saner H., Rudin B., Botros A., Pais B., Santschi V., Buluschek P., Gatica-Perez D., Urwyler P., Marchal-Crespo L. (2019). Validity of pervasive computing based continuous physical activity assessment in community-dwelling old and oldest-old. Sci. Rep..

[4] Bourgarel E., Risser C., Blanc F., Vogel T., Kaltenbach G., Meyer M., Schmitt E. (2023). Spatio-temporal gait parameters of hospitalized older patients: Comparison of fallers and non-fallers. Int. J. Environ. Res. Public Health.

[5] GAITRite Platinum Plus Classic. https://www.gaitrite.com/.

[6] Riley P.O., Paylo K.W., Kerrigan D.C., Alwan M., Felder R.A. (2008). Mobility and gait assessment technologies. Eldercare Technology for Clinical Practitioners.

[7] Kressig R.W., Herrmann F.R., Grandjean R., Michel J.P., Beauchet O. (2008). Gait variability while dual-tasking: Fall predictor in older inpatients?. Aging Clin. Exp. Res..

[8] Liu L., Stroulia E., Nikolaidis I., Miguel-Cruz A., Rincon A.R. (2016). Smart homes and home health monitoring technologies for older adults: A systematic review. Int. J. Med. Inform..

[9] Van Ancum J.M., van Schooten K.S., Jonkman N.H., Huijben B., van Lummel R.C., Meskers C.G., Maier A.B., Pijnappels M. (2019). Gait speed assessed by a 4-m walk test is not representative of daily-life gait speed in community-dwelling adults. Maturitas.

[10] Warmerdam E., Hausdorff J.M., Atrsaei A., Zhou Y., Mirelman A., Aminian K., Espay A.J., Hansen C., Evers L.J., Keller A. (2020). Long-term unsupervised mobility assessment in movement disorders. Lancet Neurol..

[11] Rubenstein L.Z., Josephson K.R. (2002). The epidemiology of falls and syncope. Clin. Geriatr. Med..

[12] Bloem B.R., Hausdorff J.M., Visser J.E., Giladi N. (2004). Falls and freezing of gait in Parkinson’s disease: A review of two interconnected, episodic phenomena. Mov. Disord..

[13] Liu Y., Zhang G., Tarolli C.G., Hristov R., Jensen-Roberts S., Waddell E.M., Myers T.L., Pawlik M.E., Soto J.M., Wilson R.M. (2022). Monitoring gait at home with radio waves in Parkinson’s disease: A marker of severity, progression, and medication response. Sci. Transl. Med..

[14] Tinetti M.E., Baker D.I., McAvay G., Claus E.B., Garrett P., Gottschalk M., Koch M.L., Trainor K., Horwitz R.I. (1994). A multifactorial intervention to reduce the risk of falling among elderly people living in the community. N. Engl. J. Med..

[15] Scheurer S., Koch J., Kucera M., Bryn H., Bärtschi M., Meerstetter T., Nef T., Urwyler P. (2019). Optimization and technical validation of the AIDE-MOI fall detection algorithm in a real-life setting with older adults. Sensors.

[16] Ganz D.A., Bao Y., Shekelle P.G., Rubenstein L.Z. (2007). Will my patient fall?. JAMA.

[17] Brodie M.A., Lord S.R., Coppens M.J., Annegarn J., Delbaere K. (2015). Eight-week remote monitoring using a freely worn device reveals unstable gait patterns in older fallers. IEEE Trans. Biomed. Eng..

[18] Botros A., Schütz N., Camenzind M., Urwyler P., Bolliger D., Vanbellingen T., Kistler R., Bohlhalter S., Müri R.M., Mosimann U.P. (2019). Long-term home-monitoring sensor technology in patients with Parkinson’s disease—Acceptance and adherence. Sensors.

[19] Khandelwal S., Wickström N. (2016). Gait event detection in real-world environment for long-term applications: Incorporating domain knowledge into time-frequency analysis. IEEE Trans. Neural Syst. Rehabil. Eng..

[20] Piau A., Mattek N., Crissey R., Beattie Z., Dodge H., Kaye J. (2020). When will my patient fall? Sensor-based in-home walking speed identifies future falls in older adults. J. Gerontol. A Biol. Sci. Med. Sci..

[21] Shah V.V., McNames J., Mancini M., Carlson-Kuhta P., Spain R.I., Nutt J.G., El-Gohary M., Curtze C., Horak F.B. (2020). Laboratory versus daily life gait characteristics in patients with multiple sclerosis, Parkinson’s disease, and matched controls. J. Neuroeng. Rehabil..

[22] Cicirelli G., Impedovo D., Dentamaro V., Marani R., Pirlo G., D’Orazio T.R. (2021). Human gait analysis in neurodegenerative diseases: A review. IEEE J. Biomed. Health Inform..

[23] ElSayed M., Alsebai A., Salaheldin A., El Gayar N., ElHelw M. Ambient and wearable sensing for gait classification in pervasive healthcare environments. Proceedings of the The 12th IEEE International Conference on e-Health Networking, Applications and Services.

[24] Stenum J., Rossi C., Roemmich R.T. (2021). Two-dimensional video-based analysis of human gait using pose estimation. PLoS Comput. Biol..

[25] Zhou H., Hu H. (2008). Human motion tracking for rehabilitation—A survey. Biomed. Signal Process. Control.

[26] Zhou L., Tunca C., Fischer E., Brahms C.M., Ersoy C., Granacher U., Arnrich B. Validation of an IMU gait analysis algorithm for gait monitoring in daily life situations. Proceedings of the 2020 42nd Annual International Conference of the IEEE Engineering in Medicine & Biology Society (EMBC).

[27] Fischer S.H., David D., Crotty B.H., Dierks M., Safran C. (2014). Acceptance and use of health information technology by community-dwelling elders. Int. J. Med. Inform..

[28] Shin G., Jarrahi M.H., Fei Y., Karami A., Gafinowitz N., Byun A., Lu X. (2019). Wearable activity trackers, accuracy, adoption, acceptance and health impact: A systematic literature review. J. Biomed. Inform..

[29] Wang J., Spicher N., Warnecke J.M., Haghi M., Schwartze J., Deserno T.M. (2021). Unobtrusive health monitoring in private spaces: The smart home. Sensors.

[30] Muresan M.P., Nedevschi S. Multimodal sparse LIDAR object tracking in clutter. Proceedings of the 2018 IEEE 14th International Conference on Intelligent Computer Communication and Processing (ICCP).

[31] Gálai B., Benedek C. Feature selection for Lidar-based gait recognition. Proceedings of the 2015 International Workshop on Computational Intelligence for Multimedia Understanding (IWCIM).

[32] Benedek C., Gálai B., Nagy B., Jankó Z. (2018). Lidar-based gait analysis and activity recognition in a 4D surveillance system. IEEE Trans. Circuits Syst. Video Technol..

[33] Yoon S., Jung H.W., Jung H., Kim K., Hong S.K., Roh H., Oh B.M. (2021). Development and validation of 2D-LiDAR-based gait analysis instrument and algorithm. Sensors.

[34] Duong H.T., Suh Y.S. (2020). Human gait tracking for normal people and walker users using a 2D LiDAR. IEEE Sens. J..

[35] Botros A., Gyger N., Schütz N., Single M., Nef T., Gerber S.M. (2021). Contactless gait assessment in home-like environments. Sensors.

[36] Gordon C.C., Churchill T., Clauser C.E., Bradtmiller B., McConville J.T., Tebbetts I., Walker R.A. (1989). Anthropometric Survey of US Army Personnel: Summary Statistics, Interim Report for 1988.

[37] Dadashi F., Mariani B., Rochat S., Büla C.J., Santos-Eggimann B., Aminian K. (2013). Gait and foot clearance parameters obtained using shoe-worn inertial sensors in a large-population sample of older adults. Sensors.

[38] Frenken T., Gövercin M., Mersmann S., Hein A. Precise assessment of self-selected gait velocity in domestic environments. Proceedings of the 2010 4th International Conference on Pervasive Computing Technologies for Healthcare.

[39] Yorozu A., Takahashi M. Development of gait measurement robot using laser range sensor for evaluating long-distance walking ability in the elderly. Proceedings of the 2015 IEEE/RSJ International Conference on Intelligent Robots and Systems (IROS).

[40] Single M., Bruhin L.C., Schütz N., Naef A.C., Hegi H., Reuse P., Schindler K.A., Krack P., Wiest R., Chan A. (2023). Development of an open-source and lightweight sensor recording software system for conducting biomedical research: Technical report. JMIR Form. Res..

[41] Bakar S.A., Hitam M.S., Yussof W.N.J.H.W. Content-based image retrieval using SIFT for binary and greyscale images. Proceedings of the 2013 IEEE International Conference on Signal and Image Processing Applications.

[42] Murtagh F., Contreras P. (2012). Algorithms for hierarchical clustering: An overview. Wiley Interdiscip. Rev. Data Min. Knowl. Discov..

[43] Single M., Bruhin L. (2023). Gait From Lidar. https://github.com/simplay/gait-from-lidar.

[44] Schober P., Boer C., Schwarte L.A. (2018). Correlation coefficients: Appropriate use and interpretation. Anesth. Analg..

[45] Müller B., Ilg W., Giese M.A., Ludolph N. (2017). Validation of enhanced kinect sensor based motion capturing for gait assessment. PLoS ONE.

[46] JudgeRoy J.O., Davis III B., Õunpuu S. (1996). Step length reductions in advanced age: The role of ankle and hip kinetics. J. Gerontol. A Biol. Sci. Med. Sci..

[47] Toro B., Nester C., Farren P. (2003). A review of observational gait assessment in clinical practice. Physiother. Theory Pract..

[48] Thong Y., Woolfson M., Crowe J., Hayes-Gill B., Jones D. (2004). Numerical double integration of acceleration measurements in noise. Measurement.

[49] Youdas J.W., Hollman J.H., Aalbers M.J., Ahrenholz H.N., Aten R.A., Cremers J.J. (2006). Agreement between the GAITRite walkway system and a stopwatch–footfall count method for measurement of temporal and spatial gait parameters. Arch. Phys. Med. Rehabil..

[50] Meldrum D., Shouldice C., Conroy R., Jones K., Forward M. (2014). Test–retest reliability of three dimensional gait analysis: Including a novel approach to visualising agreement of gait cycle waveforms with Bland and Altman plots. Gait Posture.

[51] Pantelopoulos A., Bourbakis N.G. (2009). A survey on wearable sensor-based systems for health monitoring and prognosis. IEEE Trans. Syst. Man Cybern. C Appl. Rev..

[52] Kazemi K., Laitala J., Azimi I., Liljeberg P., Rahmani A.M. (2022). Robust ppg peak detection using dilated convolutional neural networks. Sensors.

[53] Álvarez-Aparicio C., Guerrero-Higueras A.M., Rodríguez-Lera F.J., Ginés Clavero J., Martín Rico F., Matellán V. (2019). People detection and tracking using LIDAR sensors. Robotics.

[54] Haralick R.M., Sternberg S.R., Zhuang X. (1987). Image analysis using mathematical morphology. IEEE Trans. Pattern Anal. Mach. Intell..

